# The Role of Postural Restrictions after BPPV Treatment: Real Effect on Successful Treatment and BPPV's Recurrence Rates

**DOI:** 10.1155/2012/932847

**Published:** 2012-02-02

**Authors:** George X. Papacharalampous, P. V. Vlastarakos, G. P. Kotsis, D. Davilis, L. Manolopoulos

**Affiliations:** ^1^ENT Department, Elpis General Hospital, 7 Dimitsanas street, 11528 Athens, Greece; ^2^ENT Department, Lister Hospital, 64 Morecambe Close, Stevenage, Hertfordshire SG1 2BF, UK; ^3^1st Department of Otolaryngology Head and Neck Surgery, Hippocrateion General Hospital, University of Athens Medical School, 114 Vas. Sophias Avenue, 11527 Athens, Greece

## Abstract

*Background*. Canalith repositioning techniques are adequately established in the literature, as the treatment of choice for benign paroxysmal positional vertigo. However, the role of the posttreatment instructions is still not clearly defined. *Patients and Methods*. A retrospective chart review of 82 patients was conducted in order to determine the efficacy of postural restrictions, when combined with the classic canalith repositioning techniques, in terms of successful treatment and recurrence rates. Follow-up period reached at least 12 months after the initial treatment. *Results*. In this study, postural restrictions did not appear to significantly affect the outcomes of repositioning maneuvers, as well as the recurrence rate. *Conclusions*. Although this study, as well as most recent control studies, states that there is no significant effect of postmaneuver postural restrictions on both treatment and recurrence rates, larger multicentric research projects, adopting improved methodology, are still necessary in order to determine the contribution of such restrictions to both the therapeutic results and the prevention of recurrence. Adequate followup, focusing on the first six months after the initially successful repositioning maneuver, is also of paramount importance.

## 1. Introduction

Benign paroxysmal positional vertigo (BPPV) is a common peripheral vestibular disorder encountered in primary care and specialist otolaryngology and neurology clinics. BPPV is reported to comprise up to 43% of the patient population in an otology clinic [1].

Typically, BPPV is associated with a characteristic paroxysmal positional nystagmus, which can be elicited with specific diagnostic positional maneuvers [1–3]. The clinical presentation of acute vertigo after certain head movements is believed to be caused by free-floating degenerative debris in the endolymph, originating from the macula of the utricle, which moves during a head movement and gravitates into one of the semicircular canals, usually the posterior, rarely the horizontal, and more rarely the anterior semicircular canal [1, 2].

During the past 20 years, several maneuvers have been proposed for the treatment of BPPV [4–6]. Such techniques aim at returning the displaced otoconia to the utricle, so that there is no abnormal manifestation of the vestibuloocular reflexes on changing the position of the head. In the past, patients were advised to restrict posture because after a repositioning maneuver, otoconial particles floating freely inside the utricle can return to other semicircular canals, before they dissolved. That is the reason why BPPV treatment protocols, based on canalith repositioning maneuvers, traditionally included subsequent postural restrictions to prevent debris from reentering the canal [1, 2]. The patient is usually advised to avoid head and trunk movement, use a cervical collar, and sleep in a semiseated position for two days. The patient is also instructed to avoid sleeping over the affected ear for the next five days following the repositioning maneuver. The authors, who advocate such postural restrictions, argue that the period without head movements would facilitate the absorption or adhesion of otoconia to the utriculus otolithic membrane. On the other hand, authors who are against such instructions, usually state that restriction in head and trunk movements would cause some discomfort to the patients, significantly affecting their social life and behavior, while offering a totally uncertain effect on the final outcome.

The aim of the present study is to assess the efficacy of such restrictions, when combined with the classic canalith repositioning techniques. A retrospective chart review of 82 patients, divided into two different groups and treated either with canalith repositioning techniques plus postural restrictions or with repositioning maneuvers alone, was conducted. Full data from our patients was statistically analyzed in terms of final outcome, as well as recurrence rates. The follow-up period reached at least 12 months after the initial treatment. We also critically reviewed the current literature (Pubmed, Medline, and other available electronic data sources were used, along with relevant textbooks) with regard to the possible effect of postural restrictions on BPPV's treatment outcomes and recurrence rate.

## 2. Materials and Methods

Eighty-two patients suffering from BPPV, who were examined and treated at the Neurotology units of our Departments, were included in the study. There was a female preponderance, as the female-to-male ratio was 1.34/1. The range of age was 18–84 years (males: 25–84, females: 18–84), while the mean age of the patients reached 60.2 ± 12.5 years (males: 59.4 ± 11.8, females: 61.3 ± 13.7).

Patients with a clinical examination, laboratory findings, or imaging studies suggesting abnormal conditions of the central nervous system were excluded from the study. A comprehensive interview was obtained, regarding medical history, history of falls or imbalance relative to the vertigo, anxiety, onset of symptoms, and provoking factors. The Dix-Hallpike maneuver [3] was performed in all patients to diagnose posterior or anterior canal BPPV: intense vertigo in conjunction with a burst of nystagmus with the typical characteristics of latency, crescendo, fatigability, and transience was considered necessary to establish the diagnosis. On the other hand, the horizontal canal type of vertigo was diagnosed by the presence of horizontal geotropic and apogeotropic paroxysmal nystagmus provoked by turning the head from the supine to either lateral position.

The patients with posterior or anterior canal BPPV were treated by the modified Epley canalith repositioning maneuver [4], and the patients with horizontal canal BPPV were treated by the Vannucchi maneuver [6]. The appropriate maneuver was applied once, and all patients were reexamined after 7 days: in case of failure or incomplete remission of the symptoms, the same maneuver was repeated. Assessment of the success of the treatment included both the patient's report of relief from vertigo and a negative Dix-Hallpike test result. In case of a new failure, the liberatory maneuver of Semont et al. [5] was finally used.

Among patients, who visited our department from June 2008 to May 2009, forty-one (group A) followed postural restriction therapy instructions after undergoing the repositioning maneuver. The patients of group A were given instructions to follow classic postural restrictions, such as to keep their head erect and avoid sudden head movements, to wear a cervical collar for 48 hours, to sleep in a sitting position for two days, and to avoid lying on their affected side for 5 days, in order to prevent debris from going back to the affected canal.

Forty-one other patients, who visited us from June 2009 to May 2010, were also treated with the appropriate repositioning maneuver, depending on the affected semicircular canal, but were not instructed to practice postural restrictions afterwards and behave as normally as possible. These patients were designated as group B.

Follow-up care included communication by phone and, in case of recurrence of symptoms, reexamination and repetition of the repositioning procedures, according to the same plan. All patients were followed up and reevaluated by two different ENT specialists (not the physicians who performed the initial therapeutic maneuver). Follow-up period reached at least 12 months after the first attempt to treat BPPV for all patients included in this study. Reappearance of symptoms and clinical signs after the first week of the followup (and 1–3 total repositioning maneuvers performed) was considered as BPPV recurrence.

SPSS software (v.15.0), X^2^, and student's *t*-test were involved in the statistical analysis of patients' data and results. *P* values less than 0.05% were defined as statistically significant.

## 3. Results

82 patients were diagnosed and treated for BPPV with the appropriate canalith repositioning maneuver. Of the 82 patients, 34 were males and 48 females (the incidence of BPPV was about 1.34 higher in women).

Our patients were divided into two different groups (A and B). Group A included 41 patients, 17 men and 24 women, whereas another 41 patients, 18 men and 23 women, were designated as group B. The mean age of patients in group A was 58.9 ± 13.7. The mean age in group B was 60.5 ± 14.8 (Table 1). There was no significant statistical difference in age and gender ratio between the two groups.

**Figure 1:**
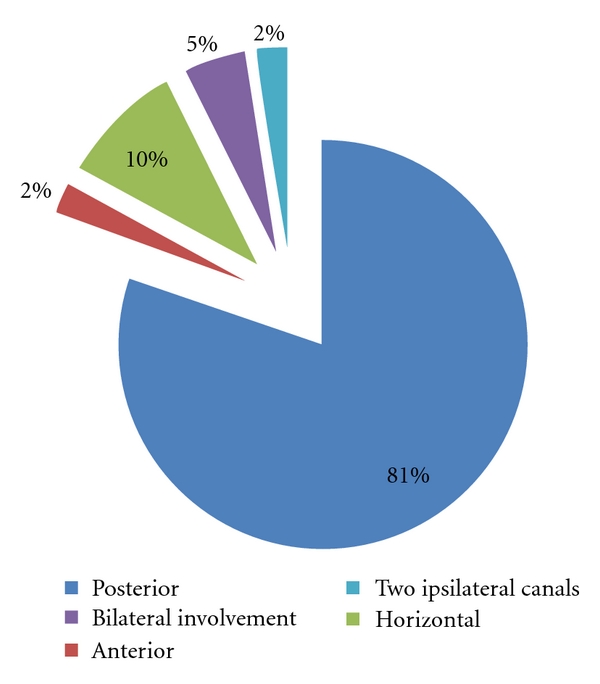
Semicircular canal involvement in our patients.

66 patients had posterior semicircular canal involvement (32 from group A and 34 from group B), while 8 (5 from group A and 3 from group B) had horizontal canal involvement, and 2 (all classified in group A) patients had the anterior canal variant. Posterior canals were affected bilaterally in 4 (2 from group A and 2 from group B) patients. In 2 patients (of group B), BPPV involving two different ipsilateral canals was identified (Figure 1). In our series, the most successfully treated BPPV appeared to be the posterior canal variant (in 30 out of 32 patients of group A and in 30 out of 34 patients of group B, complete remission of symptoms was achieved after one single maneuver). Although the number of patients was quite limited to lead to safe conclusions, the horizontal canal variant seemed quite difficult to be controlled (successfully treated after one maneuver in 1 out of 2 patients of group A and in 2 out of 5 patients of group B).

 As far as the rates of successful treatment and recurrence rates are concerned, no statistically significant difference was identified between the two groups (*P* > 0.5). Detailed data is presented in Tables 2 and 3.

## 4. Discussion

The “canalith repositioning procedure” (CRP), induced by Epley in 1992, founded a new era in the treatment of BPPV [4]. Various modifications proposed by several researchers, since Epley's original description, developed and improved repositioning procedure towards an essential and efficient therapeutic tool because of its simplicity, noninvasive nature, and apparent effectiveness in relieving vertigo [1, 2]. Therefore, CRP has progressively made BPPV the most successfully treatable cause of vertigo.

Although the efficacy of CRP as an intervention has been quite definitely established in the literature (CRP is supported to be the treatment of choice in case of BPPV by at least two recent randomized trials [7, 8]), the role of the posttreatment instructions has not been clearly defined.

Several controlled studies have been conducted to clarify this point [9]. The majority of those studies are retrospective. Most authors divide treated patients into two different groups: one group of individuals instructed to restrict their movement after CRP and another group (control group) of patients who are usually advised to behave normally at least after 48 hours from the last repositioning maneuver. Follow-up period varies between 3 and 12 months in most studies. However, only a few studies advocate follow up times longer than 6 months following CRP. Six out of the most recent seven research projects [10–15]) concluded that there is no statistically significant effect of postural restrictions on the result of repositioning maneuvers. The use of cervical collars combined with the other postmaneuver restrictions does not seem to affect the final outcomes, too [12].

The results of the present study are in accordance with the literature. In our patients postural restrictions had no statistically significant contribution to successful treatment, as treatment rates were almost similar in both groups. In addition, most of our patients, who were instructed to restrict their movement right after CRP, expressed a serious sense of discomfort. The authors believe that postural restrictions really make patients feel quite uncomfortable, leading to a “strange” behavior that could temporarily affect their social life. Important daily activities such as driving, shopping, or exercising can be quite difficult or even dangerous. Moreover, about half of our patients, classified in group A, expressed sleep disorders of some extent, mainly for the next two days following CRP, probably because of the awkward sleeping position that they were advised to adopt.

However, Cakir et al. reported that such postural restrictions enhanced the effect of canalith repositioning, when the posterior semicircular canal is involved, especially in resistant cases [16]. This recent prospective study came to a conclusion that seems to be in controversy with all other similar studies.

Some authors, who do not support the need for the traditional postural restrictions, still recommend their patients to avoid rapid head movements [13], especially for the first 48 hours after treatment. In a recent study by McGinnis et al., the authors stated that therapists could reduce the length of postural restrictions to 24 hours upright, without adversely affecting the successful result of the repositioning maneuver [11]. In our study, patients included in group B were given no posttreatment instructions at all. Normal activity was encouraged, even from the first hours after treatment. However, this fact did not affect the final result in a statistically significant level.

Most authors also support that postural restrictions do not have a statistically significant effect on recurrence rates. The results of the present study are in accordance with the current literature (Table 3), as we identified no significant difference in recurrence rates between patients who followed postural restriction after CRP (group A) and those who were advised to behave normally (group B).

The follow-up period is not clearly defined in all studies. However, most authors agree that the majority of the recurrences are experienced within the first 6 months from the initial treatment [1, 2, 4, 6]. Therefore, a follow-up period of 6–12 months is recommended to avoid underestimating recurrence rates.

Although the review of the current literature shows that the use of postural restrictions seems to be quite unjustified, there are still three crucial factors that must be taken into account, as they could lead to confusion: (a) most recent controlled studies include a relatively small number of patients, (b) the majority of the researchers do involve postural restriction of some extend (such as avoidance of head movement for 24–48 hours), even in the control group of patients, which is supposed to include only normally behaving individuals, (c) vestibular rehabilitation-central compensation is gradually taking place in all cases during the follow-up period, regardless of the treatment strategy, (d) the authors believe that the psychological effect of the instructions (probably a type of placebo effect) can crucially influence patients' ability to evaluate their condition right after CRP. This could lead them to report a subjective improvement of symptoms after postmaneuver instructions.

The results of our study are not surprising, as they are more or less in accordance with the literature. However, the authors believe that, in terms of methodology, this original research offers a valuable contribution to the scientific attempt to clarify the role of postural restriction after CRP. The crucial methodological advantages of the present study are (a) the number of patients is adequate, compared with most studies conducted and published during the 10 last years, (b) group B patients were given no postural restrictions at all, so that any probable effect gained even from restricting head movement for 24–48 hours (which is still advocated by most authors) is totally eliminated, along with the possible placebo effect of such instructions, and (c) follow-up period comprises with the gold standards, suggested by the majority of researchers. Therefore, the possibility of missing any expected recurrences is considered to be almost negligible. (d) In the present study, each semicircular canal is evaluated separately, allowing comparative assessment of CRP among the different canal variants.

Larger controlled studies (e.g., multicentric research projects), adopting suitable methodology and providing more conclusive evidence, are still necessary in order to determine the realistic contribution of postural restrictions to the final outcomes of the canalith repositioning procedure. Multiple patients' series with adequate follow-up care, as well as improved research planning, focusing on eliminating systematic errors, could lead to a consensus on the effect of postural restrictions after canalith repositioning procedure.

## 5. Conclusions

Although the efficacy of canalith repositioning maneuvers in the therapeutic management of BPPV has been definitely established in the literature, the role of the posttreatment instructions is still not clearly defined. Even though most recent control studies state that there is no significant effect of postmaneuver postural restrictions on both treatment and recurrence rates, larger multicentric studies, adopting improved methodology, are still necessary in order to determine the realistic contribution of such restrictions to the final outcomes of the canalith repositioning techniques.

## Figures and Tables

**Table 1:** Patients' groups A and B.

	Group A	Group B
Number of patients	41	41
Gender (male/female)	17/24	18/23
Age (mean ± standard deviation)	58.9 ± 13.7	60.5 ± 14.8

**Table 2:** Number of repositioning maneuvers performed.

Repositioning maneuvers performed	Group A	Group B
1	33 (80.48%)	31 (75.61%)
2-3*	2 (4.88%)	3 (7.31%)
>3**	6 (14.64%)	7 (17.08%)

*the repositioning maneuver was changed after two unsuccessful attempts

**BPPV recurred after the first week of followup.

**Table 3:** Treatment outcome and recurrences.

	Group A	Group B	
Number of patients	41	41	Level of statistical significance
Successfully treated patients (rates) after 1–3 maneuvers, no recurrence	35 (85.36%)	34 (82.92%)	*P* > 0.05
Recurrence (rates)	6 (14.64%)	7 (17.08%)	*P* > 0.05
